# Association of Dietary Polyphenol Intakes with Metabolic Syndrome and its Components: Tehran Lipid and Glucose Study

**DOI:** 10.34172/aim.28512

**Published:** 2024-07-01

**Authors:** Zohre Esfandiar, Firoozeh Hosseini-Esfahani, Parvin Mirmiran, Mitra Hasheminia, Fereidoun Azizi

**Affiliations:** ^1^Nutrition and Endocrine Research Center, Research Institute for Endocrine Sciences, Shahid Beheshti University of Medical Sciences, Tehran, Iran; ^2^Department of Biostatistics and Epidemiology, Research Institute for Endocrine Sciences, Shahid Beheshti University of Medical Sciences, Tehran, Iran; ^3^Endocrine Research Center, Research Institute for Endocrine Sciences, Shahid Beheshti University of Medical Sciences, Tehran, Iran

**Keywords:** Diet, Flavonoids, Lignans, Metabolic syndrome, Phenolic acids, Polyphenol, Stilbenes

## Abstract

**Background:**

This study was conducted to assess the prospective association between dietary polyphenols intakes and risk of metabolic syndrome (MetS) and its components.

**Methods:**

Participants in this study (n=4559) were selected from among the adults of the Tehran Lipid and Glucose Study (TLGS) with an average follow-up of 5.9+2.5 years. Biochemical and anthropometric variables were measured at baseline and follow-up examinations. A reliable and valid semi-quantitative food frequency questionnaire was used to evaluate dietary intakes. The incidence of MetS and its components in relation to polyphenols and its subclasses (phenolic acids, flavonoids, lignans, and stilbenes) was evaluated using multivariable Cox proportional hazard regression models.

**Results:**

Of the 4559 subjects who enrolled in the present study, 1765 were male aged 38.6+14.2 y and 2794 were female aged 35.9+11.7 y. The hazard ratios of MetS were 25% lower in Q2 (HR, 95% CI: 0.75, 0.64‒0.88), 22% lower in Q3 (HR, 95% CI: 0.78, 0.65‒0.94) and 24% lower in Q4 (HR, 95% CI: 0.76, 0.61‒0.95) in comparison to Q1, whereas the results for subclasses of polyphenol were non-significant. The risk of high blood pressure (BP) reduced from quartiles 1 to 4 for phenolic acid (HR: 1.00, 0.88, 0.79, 0.80, *P*_trend_=0.03). The risk of low high-density lipoprotein cholesterol (HDL-C) increased across quartiles of phenolic acid (HR: 1.00, 1.22, 1.07, 1.30, *P*_trend_=0.02).

**Conclusion:**

This study highlights the potential protective role of total dietary polyphenols in the prevention of MetS. These findings could be the starting point of upcoming trials to illuminate the optimal level of polyphenols deriving from the intake of polyphenol-rich diets to prevent MetS.

## Introduction

 Metabolic syndrome (MetS) is a group of metabolic aberrancies, including high blood pressure (BP), abdominal obesity, low high-density lipoprotein cholesterol (HDL-C) levels, hyperglycemia, and high triglycerides.^1^ The close associations between diet and MetS has been prove.^2^ Adequate findings support favorable effects of phytochemicals in plants against MetS. Among the most considerable potential phytochemicals in plants, dietary polyphenol is one of the superb candidates due to its anti-inflammatory and antioxidant properties.^3,4^

 Polyphenols are a various and large group of antioxidants contained in the foods obtained from plants. Polyphenol’s major classes have been defined with different bioavailability: phenolic acids, flavonoids, lignans, and stilbenes.^5^ Overall, there is scarce evidence about the relationship of total polyphenol and its subclasses with MetS and its components; until now, only three studies have examined the relationship between total polyphenol and its four main classes and MetS. A large cohort study showed that total polyphenol intake was associated with the incidence of MetS; however, two cross-sectional studies indicated that total polyphenols consumption did not have any association with Mets.^6-8^ As there is no concurrence among these studies, the relationship between polyphenol intake and MetS risk is still unknown. Therefore, there is a need to examine the occurrence of Mets by total dietary polyphenol intake. Our aim is to prospectively evaluate the relationship between total polyphenol intake and its four major subclasses with MetS and its components among participants of the Tehran Sugar and Lipid Study (TLGS).

## Materials and Methods

###  Study Population

**Figure 1:**
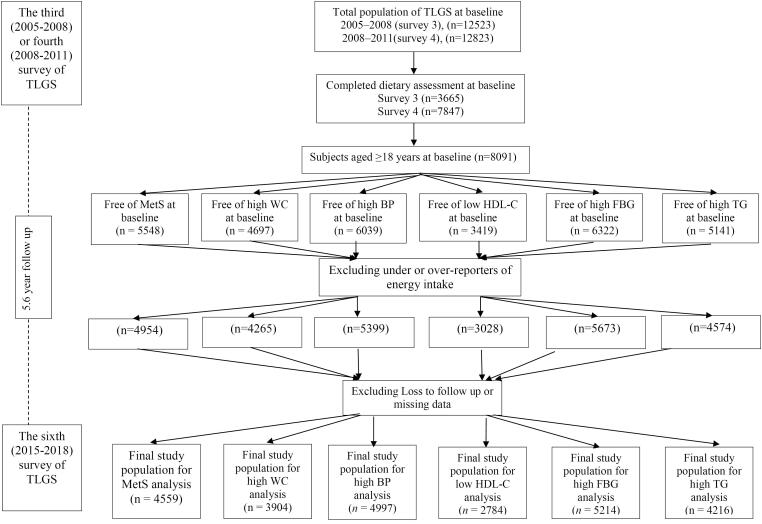
Outline of Selection of Study Participants

 TLGS is a large, prospective, community-based study to specify the risk factors for non-communicable disorders with 15,005 participants recruited in 1999 and followed every 3 years to specify recently developed diseases. A total of 8091 subjects aged ≥ 18 years, with available biochemical, anthropometric and dietary data were selected from among the participants in surveys 3 and 4 and followed until survey 6 for the current study. Exclusion criteria were: over- or under-reporting of energy intake ( ≥ 4200 or < 800 kcal/day), pregnancy or breastfeeding, and having MetS (n = 2489) at baseline. Ultimately, after excluding participants with any missing follow-up data, 4559 individuals entered the analysis. For MetS components, other independent lines were done (Figure 1). A written informed consent was signed by all participants before taking part in this investigation. All methods were performed in accordance with their relevant regulations and guidelines.

###  Measurements

 The dietary intake of the subjects was assessed using a 168-item, semi-quantitative food frequency questionnaire (FFQ). This instrument has been demonstrated to be both reliable and valid. The United States Department of Agriculture (USDA) Food Composition Table (FCT) was used whenever Iranian FCT was incomplete.^9^ The mean content values of polyphenols and its four main subclasses (phenolic acids, flavonoids, stilbenes, and lignans) in our FFQ were obtained using the Phenol-Explorer database.^5^ Details of all the measurement methods (dietary intake measurements, physical activity, anthropometric measurements and BP, and laboratory assays) are available elsewhere.^10^

###  Definitions

 MetS was determined based on the Iranian modified National Cholesterol Education Program/Adult. Having three or more of the following five risk factors is indicative of MetS^11,12^: (1) elevated BP (≥ 130/85 mm Hg) or receiving antihypertensive drug treatment; (2) high-serum TG levels (≥ 1.70 mmol/L (≥ 150 mg/dL)or receiving drug treatment; (3) impaired fasting blood serum ≥ 6.11 mmol/L (≥ 110 mg/dL) or receiving drug treatment; (4) Low-serum HDL-C (< 1.30 mmol/L (< 50 mg/dL) in women, and < 1.04 mmol/L (< 40 mg/dL) in men) or drug treatment; (5) enlarged waist circumference (WC) (WC ≥ 95 cm in women and men).

###  Statistical Analyses

 Statistical analyses were performed employing the Statistical Package for Social Sciences (version 21.0; SPSS) software. A *P* value of less than 0.05 was considered to indicate a statistically significant result. In order to compare participants’ characteristics across quartiles of energy-adjusted total polyphenol consumption, the chi-square test was used to analyze qualitative variables while the one-way ANOVA was employed for quantitative variables. In case of biochemical variables (triglyceride concentration) and non-normal nutritional, log-transformed values were used for statistical analysis. Incident MetS was defined as a dichotomous variable (yes or no) in the models. Multivariable Cox proportional hazard regression models was used for assessment of the hazards ratio (HR) and 95% confidence interval of incident MetS. Total polyphenol and main classes of polyphenol were classified into quartiles (with the first quartile considered as reference).

 The period halfway between the last follow-up visit prior to the diagnosis and the initial diagnosis visit of metabolic syndromes was identified as the event date. The duration of survival for censored participants was estimated from their initial to final visit. The participants of this study were censored as a consequence of the end of the observation period, loss to follow-up or death. In the Cox proportional hazard regression models, the overall trends of HRs from quartiles 1 to 4 for total polyphenol and its main classes were assessed using the median of each quartile. Schoenfeld’s global test of residuals was used to assess the proportional hazard assumption of multivariable Cox models. The Cox regression models were adjusted for potential confounders: baseline BMI, educational level, age, physical activity (continuous), sex, smoking status, total energy intake, total fat intakes (percentage of energy), fiber (g/1000 kcal). A *P* value < 0.20 was used for specifying inclusion in the univariable Cox regression model.

## Results

 A total of 4559 subjects participated in this study (2794 women aged 35.9 + 11.7 y and 1765 men aged 38.6 + 14.2 y). Table 1 indicates the baseline characteristics of the study population based on the quartiles of total polyphenol consumption. Subjects in the higher quartiles of total polyphenol intake had higher levels of physical activity and greater percentage of education level.

**Table 1:** Baseline Characteristics of the Study Population, Across the Quartiles of Total Polyphenol Intake in the Tehran Lipid and Glucose Study

**Characteristic**	**Total Polyphenol Consumption (mg/d)**				
	**Q1**	**Q2**	**Q3**	**Q4**	***P***
Total polyphenol intake (mg/1000 kcal/d)	653 + 183	1148 + 131	1677 + 186	2946 + 1317	
Baseline age (y)	36.0 + 12	37.0 + 12	37.6 + 12	37.1 + 12	0.88
Women, % (*n*)	59(675)	61(705)	63(721)	60(693)	0.25
Current smokers (%)	12.0	9.8	9.6	8.6	0.20
Physical activity (MET/min/wk)	1916	2101	2271	2434	0.01
Education level ≥ 14 years (%)	24	30	28	31	0.004
BMI (kg/m^2^)	25.5 + 4.1	25.7 + 4.3	25.9 + 4.6	26.0 + 4.1	0.25
Energy intake (kcal/d)	1844 + 571	2169 + 593	2475 + 603	2910 + 624	< 0.00
Carbohydrate (% of energy)	56.2 + 7.3	57.0 + 6.5	58.3 + 6.3	60.1 + 12.0	0.03
Protein (% of energy)	13.9 + 2.6	14.2 + 2.5	14.6 + 3.7	14.8 + 14.1	0.41
Total fat (% of energy)	32.0 + 7.5	31.6 + 6.2	30.6 + 5.8	30.3 + 26.2	0.27
Fiber (g/1000 kcal)	6.8 + 2.3	8.6 + 2.6	9.9 + 3	11.8 + 4.2	< 0.00
Fruits(servings/day)	1.15 + 0.67	2.05 + 1.11	2.97 + 1.55	4.86 + 3.29	< 0.00
Vegetables (servings/day)	2.39 + 1.46	3.14 + 1.71	3.05 + 2.02	4.29 + 2.55	< 0.00
Legumes (servings/day)	0.13 + 0.14	0.24 + 0.20	0.38 + 0.33	0.64 + 0.63	< 0.00
Whole grains (servings/day)	0.69 + 1.03	1.15 + 1.66	1.74 + 2.40	2.54 + 3.56	< 0.00
Refined grains (servings/day)	9.14 + 5.39	9.17 + 4.88	9.65 + 5.07	9.77 + 5.21	0.13
Dairy products (servings/day)	1.75 + 1.07	2.05 + 1.07	2.38 + 1.23	2.63 + 1.27	< 0.00
Red meat (servings/day)	0.54 + 0.63	0.67 + 0.63	0.74 + 0.70	0.78 + 0.77	< 0.00
Fish and poultry (servings/day)	1.13 + 1.04	1.25 + 1.04	1.40 + 1.28	1.70 + 1.63	< 0.00
Nuts (servings/day)	0.27 + 0.35	0.44 + 0.77	0.54 + 0.70	0.74 + 1.07	< 0.00
Tea and coffee (mL/d)	530 + 561	590 + 486	596 + 539	634 + 519	0.03
Metabolic syndrome (%)	7.9	6.5	6.8	6.8	0.01
Abdominal obesity (%)	21.8	21.1	21.9	24.7	0.15
High blood pressure (%)	10.1	9.9	11.3	11.0	0.65
Low HDL-C (%)	51.5	47.0	43.4	41.1	< 0.001
Hyperglycemia (%)	5.4	8.4	7.6	8.1	0.02
High triglyceride (%)	17.2	16.8	17.0	13.7	0.06

Q, quartiles of total polyphenol consumption; MET, Metabolic Equivalent; BMI, body mass index; MetS, Metabolic syndrome; HDL-C, High density lipoprotein cholesterol. *Values are mean + SD unless otherwise listed, †Educational level ≥ 14 years.

 Subjects in the upper quartile of energy, carbohydrate and fiber intake had higher intake of total polyphenol. Individuals in the upper quartiles of total polyphenol intake consumed more fruit, vegetable, legume, whole grain, dairy products, red meat, fish and poultry, nut, tea and coffee. The prevalence of MetS, low HDL-C and hyperglycemia were significantly different across quartiles of total polyphenol intake.

 After an average follow-up of 5.9 + 2.5 years, new-onset MetS developed in 1279 participants. The Cox proportional HRs for MetS according to the quartiles of total polyphenol and its four major subclasses are presented in Table 2.

**Table 2:** Hazard Ratios (95% CI) of Metabolic Syndrome Across Energy-Adjusted Quartiles of Total Polyphenols (Flavonoids, Phenolic Acids, stilbenes and Lignans) in Adult Participants of the Tehran Lipid and Glucose Study (n = 4559)

**Variable**	**Quartiles of Intake**				
	**Q1**	**Q2**	**Q3**	**Q4**	***P***_trend_^a^
Total polyphenols					
Crude	1	0.83(0.71‒0.97)	0.91(0.78‒1.06)	0.93(0.80‒1.09)	0.88
Model adjusted ^b^	1	0.75(0.64‒0.88)	0.78(0.65‒0.94)	0.76(0.61‒0.95)	0.09
Flavonoids					
Crude	1	0.91(0.78‒1.07)	1.00(0.86‒1.17)	0.99(0.85‒1.16)	0.58
Model adjusted ^b^	1	0.85(0.72‒1.01)	0.94(0.79‒1.13)	0.82(0.66‒1.03)	0.92
Phenolic acids					
Crude	1	1.06(0.91‒1.24)	0.90(0.77‒1.05)	1.10(0.97‒1.24)	0.31
Model adjusted ^b^	1	1.00(0.85‒1.17)	0.84(0.70‒1.00)	0.97(0.70‒1.16)	0.63
Stilbenes					
Crude	1	0.90(0.77‒1.05)	0.89(0.76‒1.04)	0.97(0.83‒1.13)	0.36
Model adjusted ^b^	1	0.98(0.84‒1.15)	0.99(0.84‒1.16)	1.09(0.99‒1.29)	0.29
Lignans					
Crude	1	1.02(0.87‒1.19)	1.01(0.86‒1.18)	1.03(0.88‒1.20)	0.84
Model adjusted ^b^	1	0.97(0.83‒1.14)	0.93(0.88‒1.10)	0.93(0.77‒1.13)	0.49

^a^Test for trend based on ordinal variable containing median value for each quartile. ^b^Adjusted for age, sex, baseline BMI, educational level, smoking status, physical activity, total energy intake, fiber, total fat intakes.

 After adjustment for potential confounders, the hazard ratio of MetS were 25% lower in Q2 [HR (95% CI): 0.75(0.64‒0.88)], 22% lower in Q3 [HR (95% CI): 0.78 (0.65‒0.94)] and 24% lower in Q4 [HR (95% CI): 0.76(0.61‒0.95)] in comparison to Q1, whereas non-significant results were found for flavonoids, phenolic acids, lignans and stilbenes intake.

 HR and 95% confidence interval of the MetS components for energy-adjusted quartiles of total polyphenol and its four major subclasses are shown in Table 3. Risk of high BP decreased from quartiles 1 to 4 for phenolic acid [HR (95% CI): 1.00, 0.88 (0.75‒1.02), 0.79 (0.67‒0.93), 0.80 (0.67‒0.95), *P*_trend_ = 0.03]. Risk of low HDL-C increased from quartiles 1 to 4 for phenolic acid [HR (95% CI): 1.00, 1.22(1.01‒1.46), 1.07(0.88‒1.30), 1.30(1.06‒1.60), *P*_trend _= 0.02].

**Table 3:** Hazard Ratios of Metabolic Syndrome Components Across Energy-Adjusted Quartiles of Total Polyphenols (Flavonoids, Phenolic Acids, Stilbenes and Lignans) Intake in Adult Participants of the Tehran Lipid and Glucose Study

**Characteristic**	**Quartiles of Polyphenol Intake**				
	**Q1**	**Q2**	**Q3**	**Q4**	***P***_trend_
Total polyphenol ^a^					
Abdominal obesity	1	0.81(0.67‒0.97)	0.97(0.79‒1.19)	0.77(0.59‒1.00)	0.38
High blood pressure	1	0.83(0.71‒0.96)	0.90(0.77‒1.05)	0.93(0.87‒1.12)	0.67
Low HDL-C	1	0.85(0.70‒1.03)	1.00(0.81‒1.24)	1.05(0.80‒1.36)	0.61
Hyperglycemia	1	1.01(0.86‒1.18)	1.03(0.87‒1.23)	0.94(0.75‒1.17)	0.39
High triglyceride	1	0.87(0.74‒1.02)	0.90(0.76‒1.07)	0.96(0.77‒1.18)	0.47
Flavonoids^a^					
Abdominal obesity	1	1.12(0.93‒1.35)	1.17(0.96‒1.42)	1.33(1.04‒1.70)	0.34
High blood pressure	1	0.93(0.80‒1.09)	0.93(0.79‒1.09)	0.93(0.77‒1.12)	0.37
Low HDL-C	1	0.79(0.65‒0.96)	0.95(0.79‒1.14)	1.02(0.81‒1.28)	0.90
Hyperglycemia	1	0.90(0.77‒1.06)	1.09(0.91‒1.29)	1.01(0.81‒1.26)	0.89
High triglyceride	1	0.66(0.73‒1.00)	0.95(0.80‒1.12)	0.90(0.73‒1.10)	0.51
Phenolic acids^a^					
Abdominal obesity	1	0.97(0.82‒1.16)	0.90(0.74‒1.10)	1.09(0.89‒1.34)	0.44
High blood pressure	1	0.88(0.75‒1.02)	0.79(0.67‒0.93)	0.80(0.67‒0.95)	0.03
Low HDL-C	1	1.22(1.01‒1.46)	1.07(0.88‒1.30)	1.30(1.06‒1.60)	0.02
Hyperglycemia	1	0.97(0.83‒1.12)	0.85(0.72‒1.00)	1.03(0.86‒1.22)	0.39
High triglyceride		0.97(0.83‒1.13)	0.94(0.80‒1.11)	1.05(0.85‒1.25)	0.14
Stilbenes^a^					
Abdominal obesity	1	1.13(0.95‒1.35)	1.16(0.97‒1.40)	1.18(0.97‒1.43)	0.94
High blood pressure	1	0.98(0.85‒1.14)	1.04(0.89‒1.21)	1.04(0.89‒1.22)	0.47
Low HDL-C	1	1.07(0.89‒1.28)	1.04(0.86‒1.25)	1.07(0.88‒1.29)	0.23
Hyperglycemia	1	1.01(0.87‒1.18)	1.12(0.96‒1.31)	1.03(0.87‒1.22)	0.92
High triglyceride		0.98(0.84‒1.14)	0.94(0.80‒1.10)	1.03(0.88‒1.22)	0.99
Lignans^a^					
Abdominal obesity	1	0.77(0.64‒0.92)	0.92(0.76‒1.10)	0.93(0.75‒1.15)	0.67
High blood pressure	1	1.05(0.90‒1.22)	0.95(0.81‒1.11)	1.04(0.87‒1.23)	0.84
Low HDL-C	1	0.99(0.82‒1.18)	0.94(0.78‒1.13)	0.82(0.66‒1.02)	0.06
Hyperglycemia	1	1.03(0.89‒1.29)	1.04(0.89‒1.22)	0.99(0.89‒1.19)	0.26
High triglyceride	1	0.99(0.84‒1.15)	0.98(0.84‒1.15)	1.01(0.85‒1.21)	0.65

^a^ Adjusted for age, sex, baseline BMI, educational level, smoking status, physical activity, total energy intake, fiber, total fat intakes.

## Discussion

 This study evaluated the association between total dietary intake of polyphenols and its individual classes and MetS and its components. The association between MetS and total polyphenol intake was found to be significant. Furthermore, a higher intake of phenolic acids was found to be inversely associated with a lower risk of high BP and a higher risk of low HDL-C.

 There is inconsistency regarding the relationship between total polyphenol intake and MetS in the past studies. Results from a large cohort of urban Polish adults is consistent toward an inverse relationship between total polyphenol intake and occurrence of MetS.^7^ However, three cross-sectional studies^6,8,13^ indicated no significant association between occurrence of MetS and total polyphenol intake; therefore, the anti-diabetic properties of total polyphenols are still unclear. Nevertheless, it is interesting to note that similar to the results of a large cohort of Polish adults,^7^ the polyphenol intake in our study was 1606 mg per day. In contrast, in the data from the cross-sectional PREDIMED study,^6^ Mediterranean countries of the EPIC Study,^14^ and non-Mediterranean countries intake of total polyphenol was considerably lower (846, 1011, and 1053 mg per day, respectively).^15^ In our study, the HR of MetS across energy-adjusted quartiles of total polyphenol was significant, although the *P* for trend was not significant. An explanation for this finding may be that the benefit of polyphenols can be obtained up to a certain point, and further consumption of these antioxidant is not effective. Thus, there might be a need for further research to determine an optimal cutoff point for benefitting from polyphenol properties.

 In line with the present study, a significant inverse association between high BP and high intake of phenolic acid has been found in the HAPIEE cohort study.^7^ Consistent with these results, individual phenolic compounds were associated with reduced risk of BP in two meta-analyses.^16,17^ Phenolic acids have shown useful effects against hypertension through their nitric oxide-mediated vasodilatory effect, antioxidant properties, and inhibiting angiotensin-converting enzyme activity.^18^

 The association between phenolic acids intake and incidence of low HDL-C was not significant in previous studies,^6-8^ whereas our results showed that the risk of low HDL-C increased from quartiles 1 to 4 for phenolic acid. In our population, white rice was the major source of phenolic acids (hydroxybenzoic acid) and the relation between lower HDL-C levels and high intake of refined carbohydrates was previously confirmed.^19^

 Important strengths of the present study include: (1) estimation of new-onset diseases without concern about reverse causality between outcomes and nutrients by the prospective design, and (2) a new insight into the association between disease and nutrients by the evaluation of nutrient consumption from numerous food sources. Limitations of this study are: (1) changes in dietary habits during follow-up, and (2) the fact that use of multiple analysis of urine or blood measures of flavonoid metabolites during the time could be more reliable than food consumption report

 In conclusion, the present study reported an inverse relation between MetS risk and total dietary polyphenol intake. These findings could be the starting point of upcoming trials to illuminate the optimal level of polyphenols deriving from the intake of a polyphenol-rich diet to prevent MetS.
